# Effectiveness of a new smartphone application on type 1 diabetes control and self-management in times of Covid-19: Randomized controlled trial

**DOI:** 10.1900/RDS.2023.19.71

**Published:** 2023-06-30

**Authors:** Nada Derkaoui, Salma Benyakhlef, Imane Rami, Ouafae El Mehraoui, Najoua Messaoudi, Hajar Charif, Nisrine Bouichrat, Imane Assarrar, Abir Tahri, Salma Derbel, Naima Abda, Siham Rouf, Hanane Latrech

**Affiliations:** 1 Department of Endocrinology-Diabetology-Nutrition, Mohammed VI University Hospital, Medical School, Mohammed the First University, Oujda, Morocco,; 2 Laboratory of epidemiology, Clinical Research and Public Health, Faculty of Medicine and Pharmacy of Oujda, Mohammed the First University, Oujda, Morocco.

**Keywords:** type 1 diabetes, self-management, glycaemic control, smartphone application, therapeutic education, telemedicine, treatment satisfaction

## Abstract

**Background:**

Self-management for type1 diabetes mellitus patients is a real challenge especially in a time of a spreading pandemic. “Ana wa Soukari” is a smartphone application for therapeutic education and insulin doses management.

**Objectives:**

Our study evaluated the effectiveness of “Ana wa Soukari” on clinical and biological outcomes of type1 diabetes self-management.

**Methods:**

This is a randomized controlled trial including 62 patients. Groupe “A” (Application users) and Group “B” (without application). Primary endpoint was HbA1c after three months’ follow-up. Secondary endpoints were number of hypoglycaemic events and treatment satisfaction which was assessed using the Arabic version of DTSQs questionnaire.

**Results:**

Sixty-two patients were included. Their mean age was 15±6,41 years. Sex ratio M/F=1,1. Mean diabetes duration was 4,9±4,3 years. All patients declared using the application at least twice a day. Mean HbA1c levels in Group A and Group B dropped from 8,3%±2,3 and 8,2%±2 respectively at baseline to 7,4%±1,5 and 8%±1,8 at three months’ follow-up. Change in hypoglycaemic episodes was –1,8±2,0(P< 0,001) for Group A and –1,2±1,5(P< 0,001) for Group B. DTSQs scores were significantly higher in group(A) than group(B).

**Conclusions:**

Self-management smartphone apps appear to be effective on glycaemic control and should be considered an adjuvant intervention to standard diabetes care.

## 1 Introduction and Background

The management of Type 1 diabetes mellitus (T1DM) is a heavy burden, not only for health workers but also for the patients themselves especially during childhood and adolescence. Dealing with a chronic disease like diabetes, requires tireless management of insulin injections, glucose monitoring, dietary habits, besides the important psychological impact of chronic diseases on their everyday life.

In fact, as soon as diabetes is diagnosed, a new relationship is created between the patient, the doctor and the family; and it is crucial to make it gradually evolving in order to improve glycaemic control and prevent degenerative complications. Introducing selfmanagement concept is the key to a perennial and sustainable glycaemic control [1].

Active interaction between T1DM patients and the attending doctor is only conceivable during a time driven consult, which may limit the amount of information and recommendations that can be given by the healthcare professional. Since March of 2020, as the pandemic of Covid -19 has spread widely in our country; telemedicine and remote care has become more a necessity than a luxury. Nowadays, free and democratized access to mobile technology and the availability of simple and secure platforms has made it possible to reinvent therapeutic education process. Numerous reviews have documented the impact of technology in enhancing diabetes self-management; besides the effectiveness of several applications regarding therapeutic education in good glycaemic control with lower acute complications prevalence [2-4].

Nonetheless, the challenge is to develop an application that meets the needs of patients according to the cultural and socioeconomic background. Our team, with the help of computer engineers and graphic designers, have imagined and designed a mobile application named “Ana wa soukari” (meaning “My diabetes and me”), in both Arabic and English, with mapped sections and virtual characters. This newly created application is fully integrated in a new pedagogical dynamism, aiming to reconcile the young T1DM patient with their disease; and overcome selfmanagement difficulties.

Through our study, we aim to determine the effectiveness on clinical and biological outcomes of T1DM self-management and evaluate treatment satisfaction after using a smartphone application that includes basic therapeutic education content (definition and types of diabetes, hypoglycaemia, hyperglycaemia…) and insulin doses management using a dosing protocol. We will present the study design and our 3 months’ follow-up results.

## 2 Materials and methods

### 2.1 The smartphone application “Ana wa soukari”

“Ana wa soukari” is a smartphone application designed and developed by a team led by the medical staff of our Endocrinology Diabetology department, with the valuable help of both communication and computer technology departments. It was released on November 2020 and counts more than 5000 users since. The application can be used on all android devices including tablets, without requiring internet connexion, with a size less than 30 MB. The development process took nearly 6 months, starting with the medical content elaboration. A group of doctors from our department reunited on a regular basis for 2 months to prepare and deliver valid evidence-based content on therapeutic education that suits T1DM patients of all ages, particularly children and adolescents. The second phase of the project was assigned to cc. All the pictures, logos and visual content were entirely shaped by the local team. Then it was converted and assembled into a practical accessible application. This final step required the involvement of all team members and especially the computer technology department.

**Figure 1.**
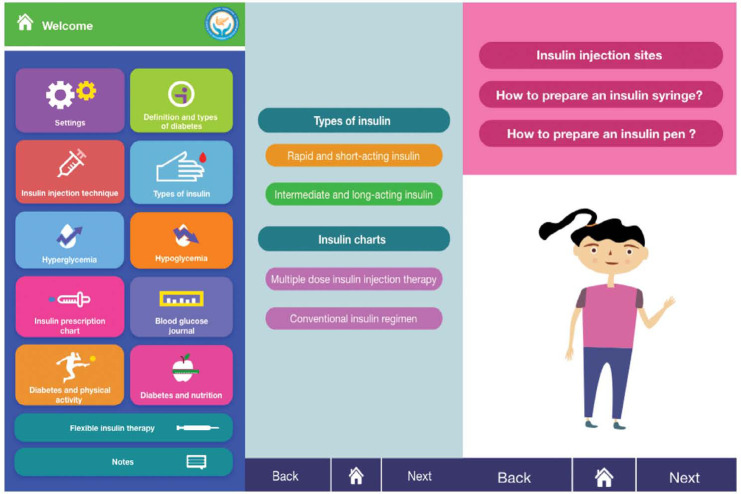
Main features and content of the application “Ana wa Soukari”

“Ana wa soukari” contains several items of the basic therapeutic education for T1DM such as hypoglycaemia and hyperglycaemia management, types and means of insulin injections, dosing adaptation according to each meal and type of physical activity. Moreover, the application offers the possibility to enter and save blood glucose levels, insulin dosages, physical activity and diet. Another main feature in our application is that it also suggests the dose of insulin to be injected taking into consideration the pre-meal blood glucose level, the patient’s usual dosing protocol and the physical activity. The ten main functions in the application are outlined in Figure 1.

### 2.2 Subjects and study plan

Our study is a randomized controlled trial. It was conducted in our endocrinology diabetology department, starting from September 2020. Eligible participants were patients diagnosed with T1DM for at least 6 months, treated with multiple daily insulin injections or insulin pumps, own and know how to use a smartphone (android), and have provided a signed consent to participate in the study. Exclusion criteria included pregnancy, illiteracy and major psychiatric disorders. Patients with major psychiatric disorders were excluded from the study due to their potential decisional capacity impairment and the difficulty to obtain their consent to participate. The total number of patients meeting the inclusion criteria of the study was 62 patients. The participants were assigned to one of the two groups using simple randomization. The application “Ana wa Soukari” was downloaded on the patients’ smartphones from Group A at baseline, and the usual care was continued afterwards for both groups including face to face follow-up visits after the first month then after 3 months. The primary study endpoint is the HbA1c level after 3 months’ follow-up. Secondary outcomes are number of hypoglycaemic events, diabetes treatment satisfaction and degenerative complications. Sociodemographic information, usability and disease related data were collected via a survey during the time of routine follow-up visits by the same group of doctors.

**Figure 2.**
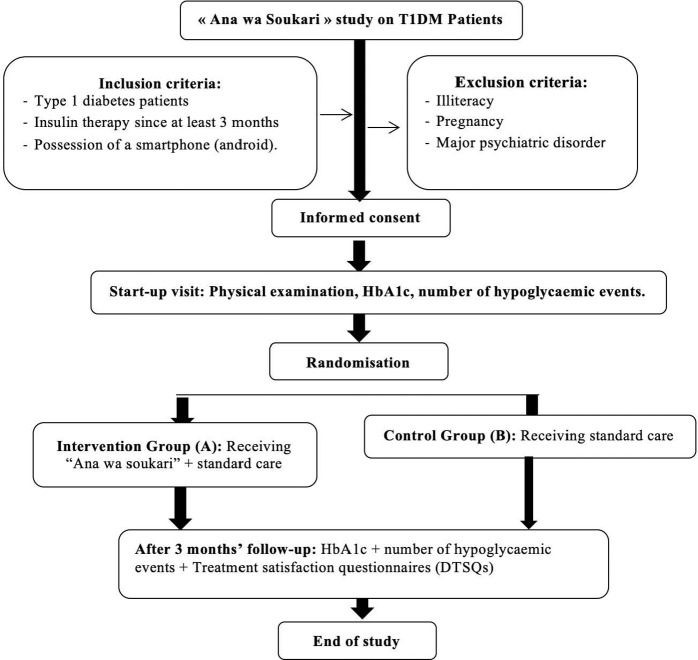
A schematic diagram with an overview of the study design and the main procedure

All parameters had been compared between two groups: Group A receiving the application + standard care, and Group B receiving the usual standard care. Figure 2 presents a schematic diagram with an overview of the study design and the main procedure.

Approval from the local ethics committee was obtained.

### 2.3 Treatment satisfaction

Diabetes treatment satisfaction questionnaire is a valid tool often used to evaluate diabetes treatment and perception of hyper and hypoglycaemia. DTSQs has been translated into more than 100 languages and is widely used in many countries, since it is internationally validated and officially approved by WHO and the International Diabetes Federation (IDF) [5]. DTSQs contains 8 items. Questions 1, 4, 5, 6, 7 and 8 assess global treatment satisfaction. Each item is scored from 6 to 0 with a higher score indicating greater satisfaction. The treatment satisfaction score can thus range from 36 (very satisfied) to 0 (very dissatisfied). The two additional items (2 and 3) measuring perceived frequency of hypo- and hyperglycemia are scored from 0 (none of the time) to 6 (most of the time) [6]. We used the Arabic version of the Adult DTSQs questionnaire that was validated in Morocco by our local team in coordination with the Health Psychology Research group (HPR)[7]. For participants under 18 years old, the Arabic version of DTSQs questionnaire is not available yet. But, since the 8 items are the same either in Teen version or Parent version, we used the same DTSQs Questionnaire for teens and parents of participants.

### 2.4 Statistical analysis

Data analysis was conducted using IBM Statistical Package for Social Sciences (SPSS) version. 28.0. Multiple variables are presented as the mean and standard deviation, frequency variables and percentages. Anova test was used for independent group comparisons in categorical variables. Comparisons between baseline and endpoint values were performed with the paired sample t-test. Pearson correlation coefficients were utilized to determine correlation values between variables. P values <0.05 were considered to be statistically significant.

## 3 Results

In total, 62 participants were included in the study. The mean age of our patients was 15 ± 6,4 years (3-30). Sex ratio M/F is 1,1 with 33 males (53%) and 29 females (47%). The average type 1 diabetes duration was 5 ± 4 years and 42 of patients (68%) had diabetes for less than 5 years. All of the participants had initially received therapeutic education in its usual form (individual and group courses with a diabetes educator, a dietician and the medical staff). Fifty-four patients (87%) were treated by multiple daily injections (MDI) including one injection of long-acting insulin in the evening and one injection of rapid or short-acting insulin before each meal. They all used insulin analogues and 8 patients (13%) were treated by insulin pumps. None of them had degenerative complications of diabetes during the study period. Usability of “Ana wa soukari” was assessed during follow up-visits. All of our patients declared using the application at least 2 times a day to enter their blood glucose and their insulin doses. Nineteen patients (59%) declared using it more than 3 times a day. Demographic, clinical and laboratory characteristics of both groups at baseline are presented in Table 1.

**Table 1.** Demographic, clinical and laboratory characteristics of both groups at baseline

Variable	Groupe A	Groupe B	P-Value
Number of patients	32	30	
Sex			
Female	13 (41%)	19 (59%)	0,324
Male	19 (59%)	14 (47%)	
Age (year)	14 ± 6	17 ± 6	0,060
Diabetes duration (year)	4 ± 4	6 ± 4	0,088
HbA1c at baseline %	8,3 ± 2,4	8,2 ± 2,0	0,860
Number of hypoglycaemic events per week	3 ± 3	3 ± 1	0,928
Diabetes treatment			
MDI	28 (87%)	26 (87%)	0,924
Insulin pump	4 (13%)	4 (13%)	

Values are presented as percentage (%) or mean ± standard deviation (SD)

There were no significant differences between the 2 groups at the beginning of the study (P>0.05). Mean HbA1c values in Group A (with application) and Group B (without application) were 8,3% ± 2,3 and 8,2% ± 2 respectively at baseline and dropped to 7,4% ± 1,5 and 8% ± 1,8 at three months’ follow-up. Although, there was no significant statistical difference between both groups regarding the change in HbA1c (HbA1c level at 3 months – HbA1c level at baseline) (P=0,07). The change in HbA1c within groups was more important and statistically significant in the group using “Ana wa soukari” than in the control group – 0,9% ± 1,7 (P= 0,016) and – 0,2% ± 0,9 (P= 0,154) respectively. The mean number of hypoglycaemic events per week was also significantly lower at endpoint for both groups. Change in hypoglycaemic episodes was – 2 ± 2 (P < 0,001) for Group A and – 1 ± 1 (P < 0,001) for Group B. HbA1c and hypoglycaemic events changes during study period are given in Table 2.

**Table 2.** HbA1c and hypoglycaemic events changes during study period

Variable	HbA1c (%)	Hypoglycaemic events / week
**Group A (Application users)**		
Baseline	8,3 ± 2,4	3 ± 3
3 months	7,4 ± 1,5	1 ± 1
P-value a	**0,016**	**< 0,00**1
**Group B (Control group**		
Baseline	8,2 ± 2	3 ± 1
3 months	8 ± 1,8	2 ± 1
P-value	0,154	**< 0,001**
**Change from baseline**		
Group A	- 0,9 ± 1,7	- 2 ± 2
Group B	-0,2 ± 0,9	-1 ± 1
P-value b	0,07	0,256

Values are mean ± SD. ^a^: Comparison of baseline and 3 months’ values within each group.

b : Comparison of mean differences between Group A and Group B within study time.

Diabetes Treatment Satisfaction Questionnaire (DTSQs) was performed in both groups at end point. Diabetes Treatment Satisfaction score was significantly higher in the group using “Ana wa Soukari” (A) than in the control group (B) with a mean score of 32 ± 3 and 28 ± 4 respectively (P < 0,001). Evaluation of the perception of hypo and hyperglycaemia was assessed through the sum of the two remaining items on the DTSQs (Questions 2 and 3). No statistically significant difference was found between the groups. The mean score of items 2 and 3 was 4 ± 2 for Group A and 3 ± 2 for Group B (P = 0,254)

Correlations between treatment satisfaction scores and outcomes of the study are presented in Table 3**.** There was a significant negative correlation between DTSQs score and the number of hypoglycaemic events (P = 0,01) which means that treatment satisfaction decreased when hypoglycaemic events’ number increased. In our study, treatment satisfaction score correlated positively with diabetes duration (P = 0.05) while there was no significant correlation between DTSQs Score and HbA1c at 3 months.

**Table 3.** Correlations between Diabetes Treatment Satisfaction score and other parameters

Variable	DTSQs Treatment satisfaction score
HbA1c at 3 months	- 0,105 (P = 0,651)
Number of hypoglycaemic events per week at 3 months	- 0,343 **(P = 0,013)**
Change of HbA1c	- 0,058 (P = 0,688)
Change of number of hypoglycaemic events/weeks	- 0,372 **(P = 0,05)**
Duration of diabetes	0,278 **(P = 0,038)**

Values are Pearson correlation coefficient (P-value).

## 4 Discussion

Incidence of T1DM is constantly increasing and tight blood glucose control and intensive support has been shown to improve glycaemic control [8] and reduce the risk of diabetes complications [9]. In addition to usual care provided for young Type 1 diabetes mellitus patients, technology-enabled diabetes self-management has proven effectiveness on glycaemic control. To the best of our knowledge, “Ana wa soukari” is the first African application for therapeutic education and self-management of T1DM in both Arabic and English. Our results have shown significant reduction in mean HbA1c levels by 0,9% ± 1,7 (P= 0,016) and in number of hypoglycaemic events by 1,8 event per week ± 2,0 (P < 0,001). Greenwood et al published a systematic review of reviews evaluating the use of smartphone applications in diabetes self-management, 18 out of 25 reviews reported significant reduction in HbA1c [4]. Four major elements emerged as essential for improved HbA1c: communication between the patients and their healthcare team, patient generated health data, therapeutic education and individualised feedback [4].. Three of which are available in our application. Often, the challenge is for the diabetes care team to get a reliable pattern of the blood glucose levels associated with insulin doses, diet and activity data, so that pertinent treatment adjustments can be made [10]. “Ana wa soukari” provides health data related to the patient including blood glucose values, meals, physical activity and other information created, recorded and eventually shared with the healthcare team. In another systematic review, Sun et al assessed the impact of using Smartphone-Based Mobile Applications on glycaemic control in adults and children with Type 1 Diabetes [11], 9 of these studies evaluated stand-alone applications, three of which showed significant improvement in glycated haemoglobin levels by 0.5%, 3 demonstrated improved adherence to glucose monitoring while one study demonstrated a reduction in hypoglycaemic events. Moreover, 5 studies evaluated a mobile app plus text-messaging/feedback system. Only one showed a significant reduction in severe hypoglycaemic events, while another single study demonstrated a reduction in median glycated haemoglobin levels by 0.3%, p<0.001[11]. In our study, scores of DTSQs were higher in the group using “Ana wa Soukari” compared with the control group. Rossi et al [12], evaluated the impact of a smartphone application interactive diabetes diary (IDD) on metabolic and weight control, time to therapy education and satisfaction with treatment in adults with type 1 diabetes over 6 months. Mean HbA1c reduction was 0,4% (P=0.68) and scores of the Diabetes Treatment Satisfaction Questionnaire (WHO-DTSQ) increased significantly over 6 months (from 26.7 ± 4.4 to 30.3 ± 4.5) (P=0.04). The use of smartphone application was effective with less time required for therapeutic education. Chatzakis et al [2], evaluated the benefits of a mobile application (Euglyca) on diabetes control and improvement of treatment satisfaction. HbA1c levels decreased by 0,9% (P<0,05) at 3 months and DTSQ scores were significantly greater at 6 months (29.1±4.0) than at baseline (26.8±5.9) (P<0.05).

In our study, the lost-to-follow-up rate was nul. Assessment of the compliance and the usability of the application was reviewed regularly with the participants during follow-up visits and they were well informed about the importance of accurate recordings. Therefore, the data collected was reliable. However, the lack of an objective assessment of usability via a special feature in the app may be one the limitations of our study. Another limitation is the lack of blinding, which could not have been avoided due to the nature of the intervention. Although ours results are promising and encouraging, the small sample size and the study period may be considered as potential limitations. Nevertheless, the use of “Ana wa Soukari” by a much larger number of patients since its uploading in 2020 is a good reflection of its many advantages. “Ana Wa Soukari” has some potentially useful features, which although not complete, they show that they may serve the population of young Type 1 diabetic patients.

### 4.1 Conclusion

Management of T1DM is a perpetual struggle. Nowadays, the global pandemic of Covid-19 has drastically changed our medical practice and our vision towards the management of T1DM, leading us to develop new tools in telemedicine. With the widespread adoption of smartphones, m-health solutions are integrated in the design of diabetes self-management education to alleviate the burden on the growing population of young type 1 diabetic patients. “Ana wa soukari” is a digital blood glucose diary and a daily assistant of rapid insulin injections that appears to be effective on glycaemic control, clinical outcomes and improvement of treatment satisfaction. Our results bring out the need for longer studies on larger populations to explore the efficacy of “Ana wa soukari” and other smartphone applications to optimize outcomes in young people with type 1 diabetes mellitus; a population that is craving for technology tools and resources.

## Abbreviations

DM: Diabetes Mellitus

EOPYY: National Organization for Health Care Services Provision (of Greece)

GIS: Geographic Information Systems

ICD: International Classification of Diseases

NCDs: Non-Communicable Diseases

NUTS: Nomenclature of Territorial Units for Statistics

T1DM: Type 1 Diabetes Mellitus

T2DM: Type 2 Diabetes Mellitus
